# Plasma-Activated Medium Inhibited the Proliferation and Migration of Non-Small Cell Lung Cancer A549 Cells in 3D Culture

**DOI:** 10.3390/ijms252413262

**Published:** 2024-12-10

**Authors:** Zhidan Sun, Chenglong Ding, Yuhan Wang, Tingting Lu, Wencheng Song

**Affiliations:** 1 Anhui Province Key Laboratory of Medical Physics and Technology, Institute of Health and Medical Technology, Hefei Institutes of Physical Science, Chinese Academy of Sciences, Hefei 230031, China; sunzhidan2022@163.com (Z.S.); dcl12041220@163.com (C.D.); 18297957335@163.com (Y.W.); 2 College of Biomedical Engineering, Anhui Medical University, Hefei 230032, China; 3 Key Laboratory for the Application and Transformation of Traditional Chinese Medicine in the Prevention and Treatment of Major Pulmonary Diseases, Anhui University of Chinese Medicine, Hefei 230012, China; 4 Collaborative Innovation Center of Radiation Medicine, Jiangsu Higher Education Institutions and School for Radiological and Interdisciplinary Sciences, Soochow University, Suzhou 215123, China

**Keywords:** plasma-activated medium, non-small cell lung cancer, proliferation, migration, 2D and 3D culture, oxidative stress, RAS/ERK

## Abstract

Lung cancer is the most common type of malignant tumor worldwide. Plasma-activated medium (PAM) is an innovative cancer treatment method that has received considerable scientific attention. The objective of this study is to evaluate the effects of PAM on the anti-tumor characteristics of non-small cell lung cancer (NSCLC) cells in two-dimensional (2D) and three-dimensional (3D) cultures. The effects of PAM treatment on the proliferative and migratory capabilities of A549 cells in 2D and 3D cultures were assessed using MTT, migration, invasion assays, and cell cycle, respectively. The study also investigated the impact of PAM treatment on the changes in the content of intracellular and extracellular reactive species and analyzed protein expression using the Western Blot method. PAM treatment inhibited the viability, migration, and invasion abilities of A549 cells in both 2D and 3D cultures, suppressed the epithelial–mesenchymal transition (EMT) process, and downregulated the expression of the RAS/ERK signaling pathway, which effectively inhibited tumor spheroid formation. Additionally, the effect of PAM on A549 cells was mediated through ROS-induced oxidative reactions, and PAM treatment exhibited greater cytotoxicity in 2D culture compared to 3D culture. As compared to 2D, the 3D cell culture model provides a viable in vitro cell model for studying the mechanisms of PAM treatment in lung cancer. PAM represents an effective new treatment for NSCLC.

## 1. Introduction

According to global cancer statistics in 2020, lung cancer is the leading cause of cancer-related mortality worldwide, accounting for approximately 18.2% of all cancer deaths [1]. Furthermore, non-small cell lung cancer (NSCLC), the most common type of lung cancer, constitutes about 85% of all lung cancer cases [2]. The heterogeneity of NSCLC poses significant challenges in treatment, as the disease is often diagnosed at an advanced stage when treatment options are limited [3], resulting in a mere 19% five-year survival rate for lung cancer patients [4]. Scientific advancements have led to substantial improvements in the prevention, diagnosis, and treatment of lung cancer. However, the prognosis for lung cancer patients is still poor due to its rapid proliferation and early metastasis [5]. Traditional treatment modalities for lung cancer include surgical resection [6], radiotherapy and chemotherapy [7], targeted therapy, and immunotherapy [8]. Surgical resection is only suitable for patients without hematogenous metastasis, and not all patients are eligible for surgery. Radiotherapy and chemotherapy continue to be the primary modalities in cancer treatment [9], but this kind of treatment has great harm to the patients. Targeted therapy and immunotherapy will lead to drug resistance mechanisms in patients, and the therapeutic effect is not optimistic [10]. Therefore, we need to find an effective treatment.

Low-temperature plasma (LTP) generates physicochemical active species primarily in the form of reactive oxygen species (ROS) and reactive nitrogen species (RNS) during room-temperature discharge processes [11]. ROS are the main biomedically effective agents of physical plasma therapy in biomedicine [12]. Consequently, LTP has been extensively applied in the biomedical field, including cancer treatment [13], blood coagulation [14], biomaterial engineering [15], and oral medicine [16], among others. There are two methods of LTP treatment for cells: direct exposure of cells to LTP and indirect treatment of cells using plasma-activated media (PAM), which is formed by first treating cell culture media with LTP. Studies have demonstrated that long-lived active species such as hydrogen peroxide (H_2_O_2_), nitrites (NO_2_^−^), nitrates (NO_3_^−^), and peroxynitrites (ONOO^−^) are the primary anti-cancer components of PAM [17,18,19]. The strength of PAM’s physicochemical properties is related to factors such as the active chemical constituents in the cell culture medium solution and the operational parameters of the plasma discharge device [20,21]. Under storage conditions of −80 °C, the anti-cancer capacity of PAM remained stable after 7 days [22]. PAM has been shown to effectively control cancer progression, such as selectively inducing apoptosis, inhibiting the viability and motility of breast cancer cells [23]. Patrakova et al. have also demonstrated that elevated ROS levels induced by PAM can enhance the cytotoxic activity against human breast cancer cell models in both two- and three-dimensions [24]. Therefore, the stability of its active species and its capability for large-scale application has led to extensive research and application of PAM in cancer treatment in recent years. PAM treatment research covers various types of cancer, including lung [25], ovarian [26], breast [23], head and neck [27], bladder [28], prostate [29], skin [30], pancreatic cancers [31], and other malignancies. Meanwhile, research has indicated that cancer cell migration is closely related to the epithelial–mesenchymal transition (EMT) process [32], and the abnormal activation of EMT can enhance cancer cell immune evasion and drug resistance and facilitate distant colonization [33]. EMT involves multiple signaling pathways, with RAS/ERK having been proven to be an important regulator of the EMT process in various cancer processes [34]. Selection of RAS/ERK signaling pathway expression to activate EMT processes has been demonstrated in Glioblastoma multiforme [35]. Furthermore, some biopharmacological and physical methods have been combined with plasma therapy to increase or synergistically enhance the treatment of cancer cells [36]. Studies have demonstrated that the combined action of PAM with doxorubicin can enhance anti-tumor activity, potentially reducing the required dosage of the chemotherapeutic agent [37]. However, whether PAM can suppress the EMT process and downregulate the RAS/ERK pathway of NSCLC cells has not yet been explored.

Good preclinical models are essential for gaining a more profound understanding of cancer cell behavior and their response to pharmacotherapy [38]. It is well known that two-dimensional (2D) monolayer culture is the most commonly used method for cell culture by researchers, but this approach has several limitations, such as the inability to simulate the complexity within the tumor microenvironment (TME) and the dynamic changes in cell-to-cell interactions [39]. In contrast, three-dimensional (3D) culture models overcome these drawbacks while maintaining the functionality, phenotypic, and molecular characteristics of the primary tumor [40]. They also enable the simulation of the TME processes like angiogenesis, immune response, proliferation, and metastasis [41,42]. Therefore, 3D culture models provide valuable research resources for replicating the TME and offer a more realistic platform for studying tumor growth, invasion, and therapeutic response. However, research on the biological and chemical effects of LTP in 3D tumor cell models is still limited. Thus, integrating 2D and 3D culture systems not only enhances the complexity of our preclinical research tools but also provides insight into the mechanisms of LTP’s effects on NSCLC cells, ultimately helping to translate LTP treatment strategies from the laboratory to clinical practice.

In this study, we employed both 2D and 3D cell culture methods to compare whether different culture conditions affect the responsiveness of A549 cells to plasma. We investigated the intrinsic mechanisms by which PAM affects the proliferative and migratory capabilities of cancer cells through Western Blot analysis, cell migration and invasion tests, cell cycle analysis, and the measurement of intracellular and extracellular ROS levels.

## 2. Results

### 2.1. Effect of Different Plasma Treatments on the Viability of A549 Cells in 2D Culture

**Figure 1:**
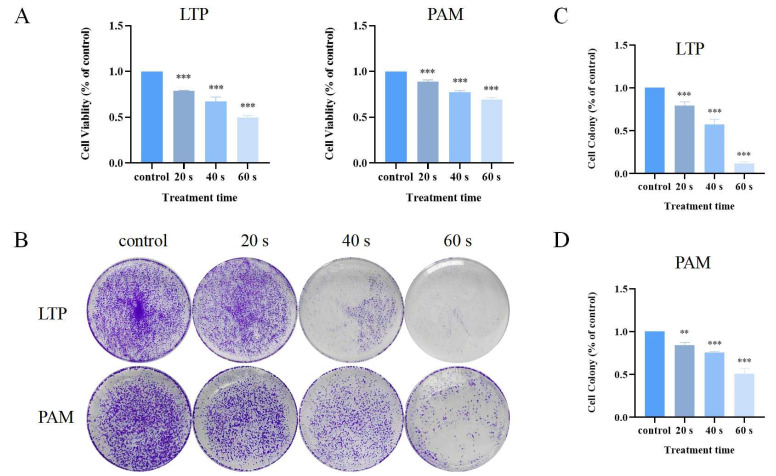
Detection of cell viability and proliferation of A549 cells in 2D culture. (**A**) Cell viability diagram of A549 cells after LTP and PAM treatment. (**B**) Colony formation images of A549 cells after LTP and PAM treatment. (**C**) Data analysis of colony formation after LTP treatment of A549 cells. (**D**) Data analysis of colony formation after PAM treatment of A549 cells. Data represent the mean ± SD of three independent experiments. ** *p* < 0.01, *** *p* < 0.001 with ANOVA compared with the control.

The effectiveness of plasma treatment methods on the viability of A549 cells is shown in Figure 1A. For A549 cells, there was a statistically significant difference in cell viability after two types of plasma treatments, and the viability of A549 cells decreased correspondingly with the prolongation of both direct and indirect treatment durations. The cell viability of A549 cells treated with LTP and PAM for 40 s was 67.1% and 77.2%, respectively. Furthermore, a colony formation experiment was performed to validate the impact of PAM treatment on cell proliferation (Figure 1B). In comparison to the control group, the two distinct plasma treatment approaches markedly suppressed the proliferation of A549 cells (Figure 1C,D). Furthermore, LTP demonstrated a more potent inhibitory effect on cell viability and proliferation compared to PAM treatment.

In accordance with previous studies, to minimize the impact of decreased cell viability on migration and invasion capabilities, we maintained the cell viability at ≥75% [43]. The PAM gradients used in subsequent experiments were 0 s, 20 s, and 40 s, respectively.

### 2.2. Effect of PAM Treatment on Viability of A549 Cells in 3D Culture

**Figure 2:**
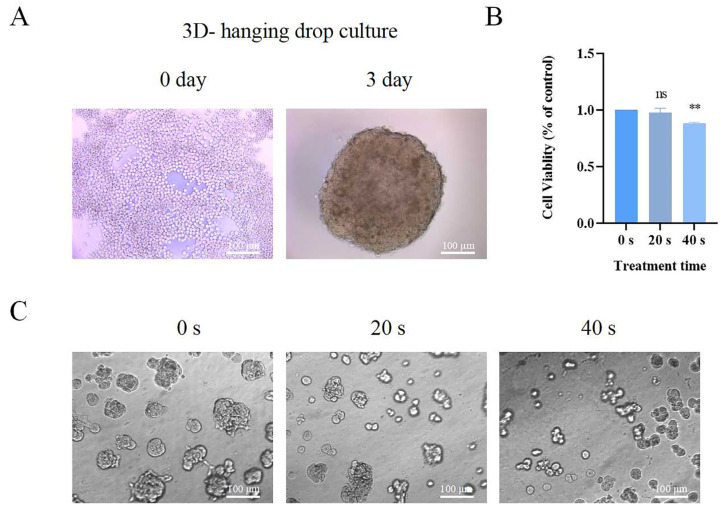
The impact of PAM treatment on the viability and growth of A549 cell spheroids. (**A**) Morphological changes of A549 cell spheroids formed by the hanging drop method over 3 days. (**B**) The effect of PAM treatment on the viability of A549 cell spheroids. (**C**) Images of spheroids formed in 3D culture based on BME after PAM treatment. Data represent the mean ± SD of three independent experiments. “ns” means no statistical difference, ** *p* < 0.01 with ANOVA compared with the control.

The formation of NSCLC tumor spheroids was evaluated using the hanging drop method. Figure 2A shows the morphology of A549 cells cultured in 3D under an inverted microscope. In 3D culture, A549 cells appeared spherical, transparent, and plump, indicating good cell condition and strong spheroid-forming capability. Concurrently, we employed the MTT assay to assess the viability of spheroid-forming A549 cells treated with PAM under 3D culture conditions. As shown in Figure 2B, in comparison to the 0 s control group, the 20 s treatment group did not exhibit any significant alterations in cell viability. In the 40 s treatment group, cell viability decreased to 87.6% of the control. It can be observed that PAM treatment can inhibit the cell viability of A549 cells in 3D culture, and cell viability shows a time-dependent decrease with extended plasma treatment durations. Additionally, compared to the viability of A549 cells cultured in 2D (Figure 1A), those cultured in 3D exhibit reduced sensitivity and increased tolerance to PAM treatment.

To observe the effect of PAM treatment on the size of spheroids formed in 3D culture, we cultivated cells in a BME-based 3D culture system, which provided a microenvironment akin to the in vivo conditions. Representative images were obtained after culturing A549 cells with PAM in the BME-based 3D culture system for 72 h (Figure 2C). The spheroid formation assay results indicated that the untreated group formed more compact tumor spheroids, while PAM treatment inhibited the tumorigenic capability. This suggests that PAM treatment can suppress the tumorigenicity of NSCLC cells in vitro.

### 2.3. PAM Treatment Inhibited Migration and Invasion in 2D and 3D Cultures

**Figure 3:**
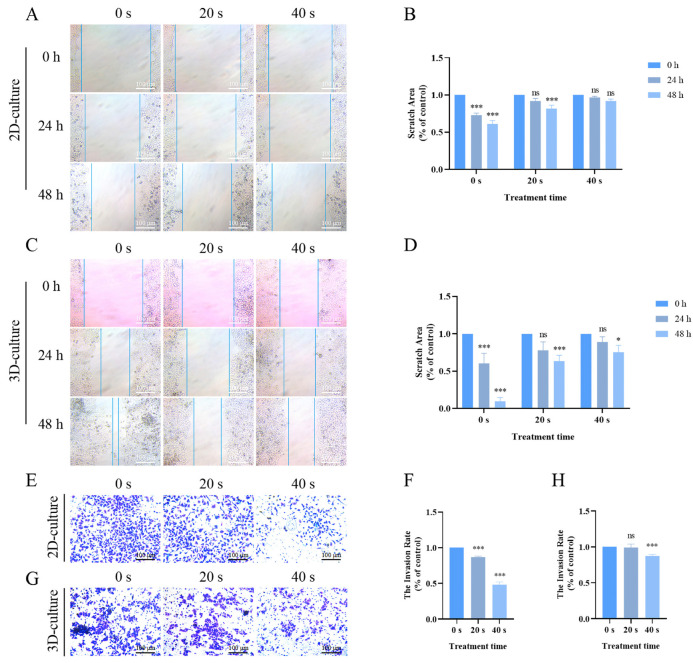
The effects of PAM treatment on the migration and invasion of A549 cells in 2D and 3D cultures. (**A**) PAM treatment inhibits the migration of A549 cells in 2D culture. (**B**) Quantitative analysis of migration data for 2D-cultured cells. (**C**) PAM treatment inhibits the migration of A549 cells in 3D culture. (**D**) Quantitative analysis of migration data for 3D-cultured cells. (**E**) Invasion images of A549 cells in 2D culture after PAM treatment. (**F**) Quantitative analysis of the number of invading cells in 2D culture. (**G**) Invasion images of A549 cells in 3D culture after PAM treatment. (**H**) Quantitative analysis of the number of invading cells in 3D culture. Data represent the mean ± SD of three independent experiments. “ns” means no statistical difference. * *p* < 0.05, *** *p* < 0.001 with ANOVA compared with the control.

Tumorigenic cells have the capacity to spread and multiply. To assess the impact of PAM treatment on A549 cells in both 2D and 3D cultures, we conducted migration and invasion assays. Compared to the 0 s control group, cells treated with PAM exhibited reduced migratory capacity, while cells cultured in 3D demonstrated faster migration compared to the 2D cultured cells of the PAM group (Figure 3A,B). Subsequently, we conducted invasion assays with Matrigel and observed that the number of invading cells in the PAM-treated groups, in both 2D and 3D culture settings, was significantly reduced when juxtaposed with the 0 s control group (Figure 3C,D). Our results indicate that PAM treatment inhibits both migration and invasion in A549 cells across 2D and 3D culture models.

### 2.4. PAM Treatment Increased Reactive Species Production in 2D and 3D Cultures

**Figure 4:**
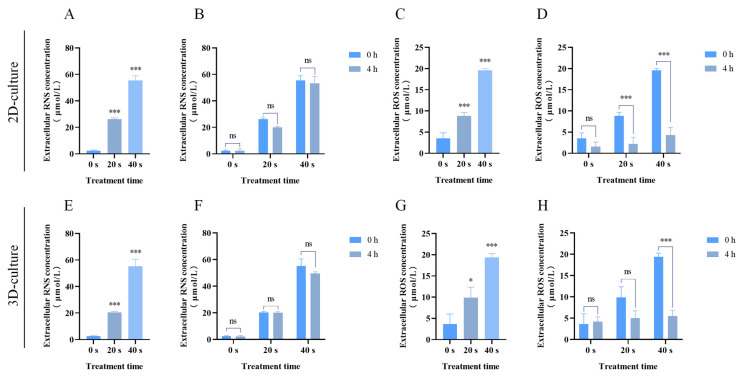
Extracellular RONS levels in PAM-treated A549 cells in 2D and 3D cultures. (**A**) Extracellular NO concentration of A549 cells in 2D culture at 0 h of PAM treatment. (**B**) Extracellular NO concentration of A549 cells in 2D culture at 0 h and 4 h of PAM treatment. (**C**) Extracellular H_2_O_2_ concentration of A549 cells in 2D culture at 0 h of PAM treatment. (**D**) Extracellular H_2_O_2_ concentration of A549 cells in 2D culture at 0 h and 4 h of PAM treatment. (**E**) Extracellular NO concentration of A549 cells in 3D culture at 0 h of PAM treatment. (**F**) Extracellular NO concentration of A549 cells in 2D culture at 0 h and 4 h of PAM treatment. (**G**) Extracellular H_2_O_2_ concentration of A549 cells in 2D culture at 0 h of PAM treatment. (**H**) Extracellular H_2_O_2_ concentration of A549 cells in 2D culture at 0 h and 4 h of PAM treatment. Data represent the mean ± SD of three independent experiments. “ns” means no statistical difference. * *p* < 0.05, *** *p* < 0.001 with ANOVA compared with the control.

**Figure 5:**
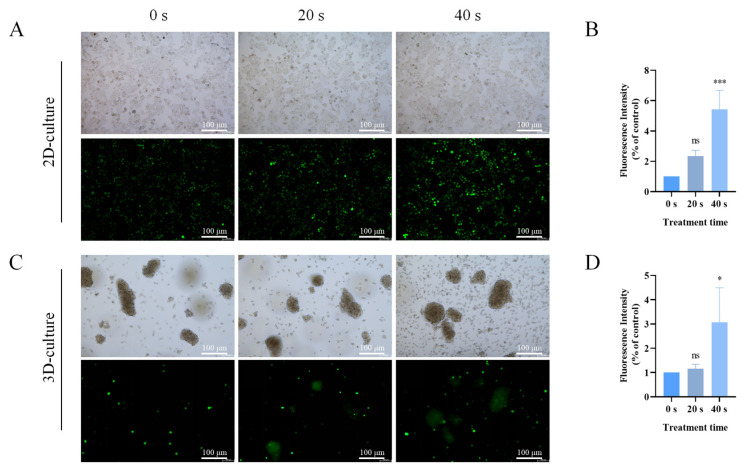
Intracellular ROS levels in PAM-treated A549 cells in 2D and 3D cultures. (**A**) Morphology and intracellular ROS fluorescence images of A549 cells in 2D culture after 4 h of PAM treatment. (**B**) Quantification of intracellular ROS in 2D-cultured A549 cells. (**C**) Morphology and intracellular ROS fluorescence images of A549 cells in 3D culture after 4 h of PAM treatment. (**D**) Quantification of intracellular ROS in 3D-cultured A549 cells. Data represent the mean ± SD of three independent experiments. “ns” means no statistical difference. * *p* < 0.05, *** *p* < 0.001 with ANOVA compared with the control.

Extracellular active substances mainly include ROS and RNS. As shown in Figure 4, in both 2D and 3D culture models, the concentration of RNS increased in a positively correlated manner with prolonged PAM exposure times (Figure 4A,E), but no significant difference was observed between the 4 h and 0 h time points (Figure 4B,F). Concurrently, as depicted in Figure 4C,G, extracellular ROS levels also increased in a positively correlated manner with extended PAM exposure times. Compared to the 0 h time point, extracellular ROS levels paradoxically decreased at the 4 h time point, and this difference was statistically significant (Figure 4D,H). This decrease may be attributed to the high fluidity and permeability of the cell membrane, allowing highly permeable ROS to enter the cell interior more readily [44]. Therefore, we further examined the levels of ROS in 2D and 3D cells cultured. The results, as shown in Figure 5, indicated that after 4 h of PAM treatment, intracellular ROS levels were elevated relative to the control group and escalated in a correlated manner with prolonged PAM exposure times. These results imply that treatment with PAM leads to an increase in ROS accumulation within A549 cells across both 2D and 3D culture models, which may consequently activate intracellular oxidative stress responses.

### 2.5. Effects of NAC Treatment in 2D and 3D Cultures

**Figure 6:**
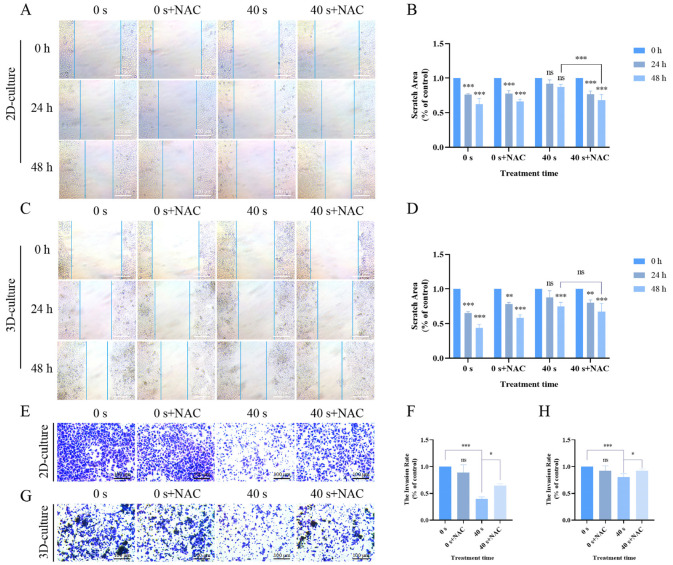
ROS validation experiments with 0 s, NAC+0 s treatment, 40 s PAM treatment, and 40 s+NAC treatment groups. (**A**) Migration assay of 2D-cultured cells. (**B**) Quantitative analysis of cell migration in 2D culture. (**C**) Migration assay of 3D-cultured cells. (**D**) Quantitative analysis of cell migration in 3D culture. (**E**) Invasion images of A549 cells cultured in 2D after PAM treatment. (**F**) Quantitative analysis of invading cells in 2D culture. (**G**) Invasion images of A549 cells cultured in 3D after PAM treatment. (**H**) Quantitative analysis of invading cells in 3D culture. Data represent the mean ± SD of three independent experiments. “ns” means no statistical difference. * *p* < 0.05, ** *p* < 0.01, *** *p* < 0.001 with ANOVA compared with the control.

In previous experiments, we observed that PAM treatment could induce the accumulation of intracellular ROS, which is associated with oxidative stress. To delve deeper into this effect, we added the active form of NAC, which is a ROS scavenger, to pretreat the A549 cells. We found that compared to the 0 s group, the migration and invasion capabilities of the PAM-treated group were significantly restored, indicating that the addition of NAC ameliorated the suppressive effects of PAM on the malignant behavior of A549 cells (Figure 6). Therefore, our experimental data indicate that the supplementation with NAC substantially reversed the malignancy of A549 cells, which was initially triggered by an increase in ROS levels.

### 2.6. PAM Treatment Blocked Cell Cycle in 2D and 3D Cultures

**Figure 7:**
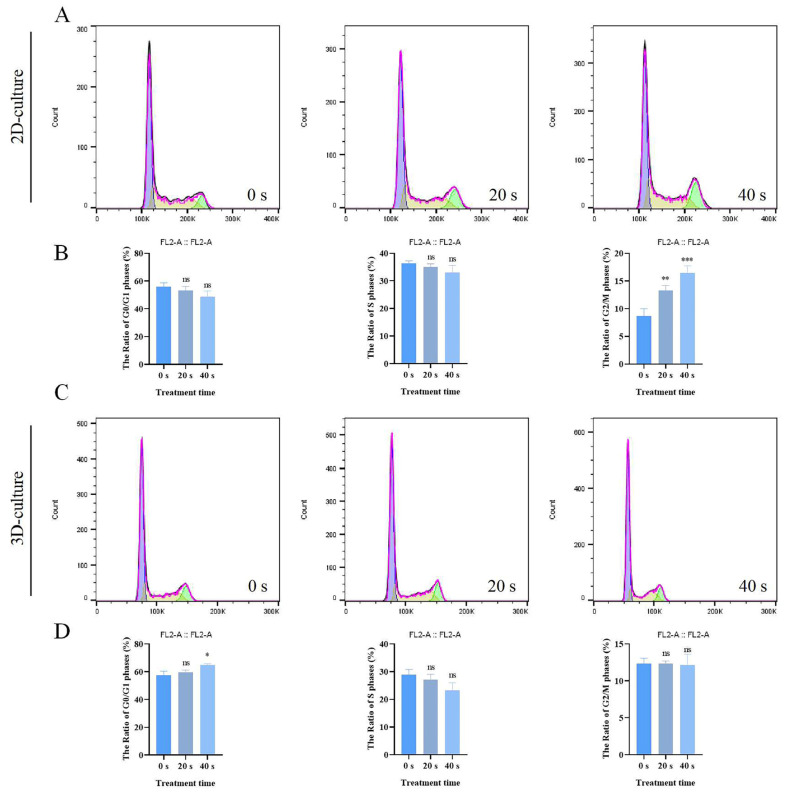
Impact of PAM treatment on the cell cycle of A549 cells in 2D and 3D cultures. (**A**) Flow cytometric analysis of the cell cycle distribution of A549 cells treated with PAM in 2D culture, where the purple region represents the G0/G1 phase, the green region represents the S phase, and the red region represents the G2/M phase. (**B**) Quantification of the percentage of cells in the G0/G1, S, and G2/M phases following PAM treatment in 2D culture. (**C**) Cell cycle distribution of A549 cells treated with PAM in 3D culture. (**D**) Quantification of the percentage of cells in the G0/G1, S, and G2/M phases following PAM treatment in 3D culture. Data represent the mean ± SD of three independent experiments. “ns” means no statistical difference. * *p* < 0.05, ** *p* < 0.01, *** *p* < 0.001 with ANOVA compared with the control.

The impact of PAM on the A549 cell cycle was assessed using flow cytometry. As shown in Figure 7, under the 2D culture condition, the G2/M phase ratio of A549 cells increased in a correlated manner with the duration of PAM treatment, indicating that PAM can significantly potentiate cell cycle arrest at the G2/M phase. In the 3D culture model, however, treatment with PAM caused a higher percentage of cells to accumulate in the G0/G1 phase. In summary, our experimental results demonstrate that PAM possesses the capacity to inhibit A549 cell proliferation and affect specific stages of the cell cycle process under various culture conditions.

### 2.7. PAM Treatment Inhibited RAS/REK Pathway and the EMT Process

**Figure 8:**
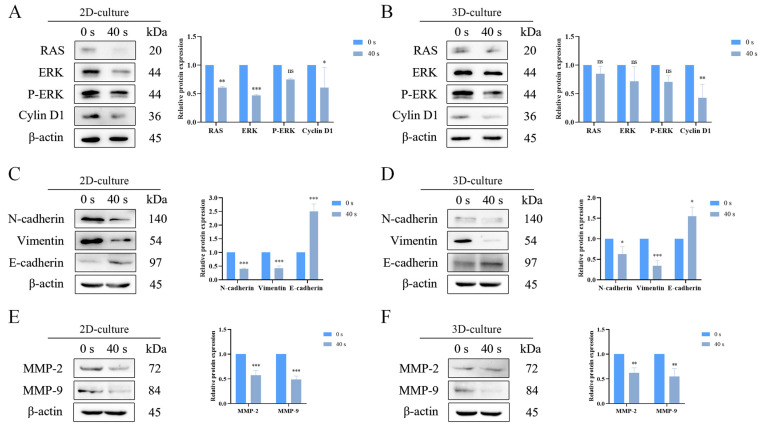
PAM treatment downregulates RAS/ERK, EMT process, and migration protein expression. (**A**) Analysis of RAS, ERK, p-ERK, and Cyclin D1 proteins expression in 2D-cultured A549 cells, with β-actin as the control. (**B**) Analysis of RAS, ERK, p-ERK, and Cyclin D1 proteins expression in 3D-cultured A549 cells, with β-actin as the control. (**C**) Analysis of N-cadherin, vimentin, and E-cadherin protein expression in 2D-cultured A549 cells, with β-actin as the control. (**D**) Analysis of N-cadherin, vimentin, and E-cadherin protein expression in 3D-cultured A549 cells, with β-actin as the control. (**E**) Analysis of MMP-2 and MMP-9 protein expression in 2D-cultured A549 cells, with β-actin as the control. (**F**) Analysis of MMP-2 and MMP-9 protein expression in 3D-cultured A549 cells, with β-actin as the control. Data represent the mean ± SD of three independent experiments. “ns” means no statistical difference. * *p* < 0.05, ** *p* < 0.01, *** *p* < 0.001 with *t*-test and ANOVA compared with the control.

This study utilized Western Blot analysis to assess the levels of proteins associated with the RAS/ERK signaling pathway, EMT markers, and MMPs in A549 cells treated with PAM in both 2D and 3D cultures. Figure 8 demonstrates that PAM treatment downregulated the protein expression levels of RAS, ERK, p-ERK, N-cadherin, vimentin, matrix metalloproteinase-2 (MMP-2), and matrix metalloproteinase-9 (MMP-9) in NSCLC cells while upregulating the protein expression level of E-cadherin. These results suggest that PAM treatment inhibits the RAS/ERK signaling pathway, the EMT process, and the expression of MMP proteins in A549 cells. This study, therefore, illustrates that PAM treatment suppresses the RAS/ERK signaling pathway and EMT process, as well as the expression of MMP proteins in A549 cells across 2D and 3D culture conditions.

## 3. Discussion

In recent years, PAM has been extensively studied and is considered a candidate for the treatment of various cancers. Previous studies have demonstrated that PAM can inhibit the growth of multicellular tumor spheroids in colon adenocarcinoma and have confirmed that H_2_O_2_ plays a pivotal role in inducing DNA damage [22]. PAM may induce cell death in metastatic melanoma and pancreatic cancer cells by activating the innate immune system [31]. PAM, acting as a donor of ROS and its derived H_2_O_2_, leads to apoptosis in A549 cells by inducing a spiral apoptotic cascade involving the mitochondrial–nuclear network [45]. PAM can inhibit the in vitro and in vivo metastasis of ovarian cancer cells by blocking the activation of the mitogen-activated protein kinase (MAPK) pathway and reducing MMP-9 secretion [26]. Simultaneously, studies have shown that PAM treatment also exerts anti-tumor effects against chronic chemoresistant ovarian cancer cells both in vitro and in vivo [46]. Furthermore, PAM has been proven to be more effective at killing bladder cancer cells than drugs that induce apoptosis [28]. However, the majority of these investigations have been carried out using conventional 2D culture systems. Due to the fact that 3D culture models provide an assessment system closer to the in vivo environment, we utilized the hanging drop method to allow cells to self-organize into compact spheroids under the influence of gravity, mimicking the 3D environment of in vivo cell growth. We explored PAM’s impact on NSCLC across 2D and 3D cell culture models. Findings revealed that PAM decreased A549 cell viability in both culture systems (Figure 1 and Figure 2). Notably, PAM displayed a more pronounced cytotoxic effect on cells cultured in 2D conditions relative to those in 3D conditions. Furthermore, PAM treatment effectively suppressed the tumorigenicity of spheroids in 3D culture (Figure 2). This is consistent with a previous study of PAM inhibiting the viability of head and neck cancer cells [27]. The differences in response to PAM between 2D culture and spheroids may be due to the fact that PAM must diffuse within the spheroid to damage cells, whereas in 2D culture, PAM is in direct contact with the cells and can more easily interact with the cell membrane, potentially leading to more direct cytotoxicity. Therefore, the differences in cytotoxicity of PAM in different culture models are not only dependent on the exposure time of the cell culture medium to LTP but also on the cells’ self-organization capabilities [47].

Cancer cells are characterized by their high migration and proliferation capabilities. Through migration and Matrigel invasion assays, we found that PAM treatment effectively inhibits the migration and invasion capabilities of A549 cells in both 2D and 3D culture conditions (Figure 3). ROS are important products of cellular activity, which are generated regularly during metabolism and involved in the regulation of signal transduction cascades. Additionally, cancer cells have higher metabolic activity compared to normal cells, thus possessing a higher basal ROS level at steady-state conditions [48]. When cells are treated with an optimal dose of PAM, cancer cells are more susceptible to ROS-induced oxidative stress while minimizing damage to normal cells [49,50]. Excessive RNS and ROS necessarily produce numerous potential cytotoxic and genotoxic by-products, thereby causing cellular damage, oxidative stress, and inflammatory responses [13,19,24,31,51]. Previous studies have indicated that high-dose PAM, containing excessive ROS and RNS, may increase the toxicity in lung cancer cells, leading to mitochondrial dysfunction and ferroptosis [52]. Therefore, in the present study, our experimental design was informed by previous research and preliminary experimental outcomes, employing a gradient of LTP treatment for 0–60 s and PAM exposure to LTP for 0–60 s. Ultimately, we decided to cap the PAM exposure at a maximum of 40 s to maintain cell viability at ≥75%. This approach aimed to explore the effects of PAM on cell proliferation and migration while avoiding the potential cytotoxic effects that could result in cell death due to high-dose PAM exposure. At the same time, this study explored the effects of PAM treatment on the levels of RNS and ROS within A549 cells across both 2D and 3D culture systems, and the results indicated that the concentrations of both substances increased with prolonged exposure to PAM (Figure 4). However, the extracellular H_2_O_2_ concentration in A549 cells rapidly declined after 4 h of culture. Moreover, we observed a rapid increase in intracellular ROS levels after treating A549 cells with PAM for 4 h (Figure 5). These results suggest that the H_2_O_2_ component in PAM may be a primary factor contributing to the increased intracellular ROS levels and the induction of redox reactions in A549 cells. This is consistent with findings from previous studies [53]. Previous research has demonstrated that these exogenous ROS generated by LTP penetrate into cells and induce increased oxidative stress [22]. To further verify the role of ROS in the anti-tumor effects of PAM, we investigated the impact of adding NAC on the migration and invasion behavior of PAM-treated cancer cells. The experimental results showed that the addition of NAC substantially reversed the malignant behavior of A549 cells (Figure 6), confirming that ROS play a central role in governing the migration and invasion of cancer cells. Additionally, although H_2_O_2_ is a known effective active ingredient, other active substances within the entire PAM composition may act in concert with H_2_O_2_ to produce a synergistic effect [54]. Previous studies have suggested that the growth-inhibiting effects of PAM on 2D monolayers and 3D spheroids may not be solely attributed to H_2_O_2_ but also to the collaborative involvement of other chemical constituents within PAM [27]. Bauer et al. also demonstrated that the long-lived active substances in PAM, such as H_2_O_2_ and nitrite, act synergistically to induce apoptosis in tumor cells [18]. The complexity of PAM offers a broader spectrum of reactive species that act synergistically to exert the observed biological effects, which may not be fully replicated by H_2_O_2_ alone. Therefore, it is necessary to further investigate the other primary active components of PAM and how they collectively influence the efficacy of cancer treatment in future studies.

Previous research has shown that PAM can specifically elevate ROS levels in androgen receptor (AR)-independent prostate cancer cells, which in turn suppresses cell migration, triggers G0/G1 phase cell cycle arrest and promotes apoptosis [29]. Furthermore, it has been demonstrated that PAM can cause cell cycle arrest in human skin melanocytes by modulating the G0/G1 and G2/M phases, thus slowing down the progression of skin lesions [55]. These findings suggest that PAM treatment can affect cell cycle progression. Our study shows that PAM treatment leads to the retention of cells in the G2/M phase under 2D culture conditions, while in 3D culture conditions, it causes cells to remain in the G0/G1 phase (Figure 7). This indicates that PAM treatment can influence the cell cycle process under different culture conditions.

The differential cell cycle phase arrest induced by PAM treatment in A549 cells under 2D and 3D culture settings may be attributed to the smaller surface area of contact between spheroids and the culture medium in 3D culture, leading to variations in the permeability and efficacy of PAM treatment. Additionally, cells cultured in 3D may exhibit enhanced antioxidant capacity, which could be related to the complex structures formed in 3D culture. These structures might provide an additional protective mechanism, thereby reducing the cells’ sensitivity to PAM treatment. Previous studies have also indicated that compared to 2D culture, cancer cell lines in 3D culture exhibit reduced sensitivity to drugs due to increased interactions between cells and the extracellular matrix (ECM) [56].

The EMT is a process by which individual cells with epithelial architecture can transform into motile and invasive mesenchymal phenotype cells. Mesenchymal cell migration is one of the primary modes of cancer cell migration [57]. E-cadherin is a marker for the maintenance of adherens junctions, while N-cadherin and vimentin are associated with the mesenchymal cell state. At the beginning of EMT in tumor cells, adherens junctions are disrupted, the type of cadherin molecules is switched, and the cytoskeleton undergoes reorganization to enhance motility [58]. ECM regulates cellular physiology, growth, survival, differentiation, and movement [59]. MMP-2 and MMP-9 are among the most widely studied biomarkers of the MMPs family, which can degrade the ECM and basement membrane of tumor cells, thereby facilitating cancer cell migration and invasion [60]. Consequently, cell migration is a complex process involving the interaction of multiple factors. The ECM provides physical support and chemical cues, MMPs modulate ECM degradation, and EMT endows cells with the capacity for migration and invasion. These elements work in concert to drive the migration and invasion of cancer cells [57,58,59,60].

Mechanistically, the MAPK pathway is a highly conserved signaling pathway across evolution that is involved in a multitude of biological processes, including proliferation, migration, invasion, metastasis, and survival. Among these, the RAS/ERK subpathway is one of the classic pathways within the MAPK cascade [61]. Mutations in the RAS gene are most commonly observed in NSCLC patients [62]. Previous studies have indicated that the abnormal activation of the RAS/ERK pathway can promote tumor cell proliferation and migration [63], and LTP treatment induces neurodifferentiation through the activation of the RAS/ERK signaling pathway [64]. Therefore, targeting the RAS signaling pathway is important for the treatment of NSCLC. However, the regulatory effects of PAM treatment on NSCLC cells within the RAS/ERK pathway remain unknown. Our Western Blot analysis revealed that PAM treatment downregulated the expression levels of RAS, p-ERK, and Cyclin D1, inhibited the EMT process, as well as the expression of extracellular matrix enzymes MMP-2 and MMP-9, indicating that PAM treatment can affect NSCLC cells function through these pathways (Figure 8). Previous research has established that the RAS/ERK signaling cascade plays a pivotal role in modulating cell cycle advancement through the downregulation of Cyclin D1 expression [65], and RAS-dependent MAPK cascades are the primary signaling pathways inducing epithelial–mesenchymal movement and invasiveness [35]. Activation of the RAS/ERK pathway can induce the occurrence of EMT in bladder cancer through various mechanisms, affecting the expression and function of N-cadherin, vimentin, and E-cadherin [66]. Therefore, the interplay between the RAS/ERK pathway and N-cadherin, vimentin, and E-cadherin, as well as MMP-2 and MMP-9, is crucial for cancer cell proliferation and migration [67]. PAM contains reactive species such as ROS and RNS, which play pivotal roles in the TME [51]. These reactive species may interact with biomembranes to produce various functional modifications, thereby triggering intracellular signaling cascades [68,69]. Magazzù et al. have shown that the application of AFM and OT techniques has provided profound insights into the mechanical interactions between cells and matrices, particularly in understanding the impact of ECM stiffness on cellular behavior [70]. This resonates with the findings of Park et al. in NSCLC cells, where they highlighted the regulatory role of ECM stiffness on cell proliferation and PD-L1 expression [71]. Given that the microenvironment in which tumor cells reside typically exhibits varying degrees of matrix stiffness, the increase in ROS may alter the mechanical properties of cells through oxidative stress, affecting the cytoskeleton and the cross-linking of the ECM, thereby leading to drastic changes in cellular matrix stiffness [72,73,74]. Such alterations could further impact the migratory and invasive capabilities of cells, as well as their response to treatment [75]. Moreover, studies indicate that ROS can inhibit the RAS/ERK signaling pathway via oxidative stress responses, thereby regulating the proliferation and migration of cancer cells [76]. This suggests that in both 2D and 3D cultures, PAM inhibits the proliferation and migration of A549 cells by blocking the RAS/ERK pathway.

In 3D cell culture systems, cells often reside in various cell cycle phases due to the presence of oxygen and nutrient gradients, which more closely mimic the in vivo cellular state [77]. Compared to 2D culture, cells in 3D culture exhibit more physiological characteristics in vivo, including distinct responses to drugs and external stimuli [78]. Moreover, the limited permeability of the culture medium in 3D culture may affect cell viability and protein expression. Cells at the periphery of the spheroids are metabolically active and typically possess the highest invasive and proliferative capabilities [79]. It may lead to differences in the performance of PAM treatment on cancer cells between 2D and 3D culture systems. However, since this similar situation also exists in vivo, the results obtained from 2D models should be extrapolated to in vivo models with caution in future studies. Although we employed 3D culture models to mimic the TME, these models still fall short of fully replicating the complexities within tumors. Future research should consider conducting studies with larger sample sizes and a diversity of models to enhance the universality and reliability of our findings. Due to the constraints of our laboratory’s resources and conditions, we were unable to test the effects of chemotherapy drugs in monotherapy and combination therapies within this study. To enhance the preclinical value of the research, we plan to explore the potential of PAM in combination with chemotherapy drugs in future studies, aiming to overcome the limitations of the current research and provide more valuable insights for the clinical treatment of NSCLC.

## 4. Materials and Methods

### 4.1. Cell Culture

Human NSCLC A549 cells were employed in this study, which were derived from the Chinese Academy of Sciences Typical Culture Preservation Committee cell bank. Cells were inoculated into DMEM medium (DMEM, high glucose, Sangon Biotech, Shanghai, China) containing 10% fetal bovine serum (FBS, Sangon Biotech, Shanghai, China) and 1% penicillin/streptomycin (Sangon Biotech, Shanghai, China), incubated in a 37 °C incubator (Thermo Fisher Scientific, Waltham, MA, USA) containing 5% CO_2_. As cells reached around 75–80% of the fusion surface, the cells were treated with PAM, which was prepared with LTP for 0 s, 20 s, 40 s, and 60 s.

A549 cells were cultured in 3D technology by the hanging drop method [80]. When the cells grew well, they were digested with trypsin (Sangon Biotech, Shanghai, China) to make a cell suspension. This suspension was added to the Petri dish cover, with 5000 cells/drop and a volume of 30 μL. A 3 mL PBS solution was added to the bottom of the culture dish to keep the humidity needed for cell growth. Culture was maintained for 3 days, and the follow-up experiment was continued after the cells were aggregated into spheres.

### 4.2. Experimental Device

In this study, a plasma dielectric barrier discharge device was used, which was introduced by Zhou et al. [81]. In this experiment, helium (He, 99.999%) was applied in the discharge procedure, characterized by an effective voltage of 3.78 kV, a frequency of 25 kHz, and a helium flow rate set at 1.2 L/min. When using this device to prepare PAM, it is necessary to keep the device closed, ventilate for 90 s, and subsequently process the medium samples according to the time gradient 0 s–60 s.

### 4.3. Cell Viability Assay

The assay involving 3-(4,5-dimethylthiazol-2-yl)-2,5-diphenyltetrazolium bromide (MTT, Sigma-Aldrich, St. Louis, MO, USA) was employed to ascertain the influence of PAM on cellular vitality. When A549 cells with stable passage were cultured to 75%–80% fusion, the cells were treated with PAM for 24 h. Following this, the cells were incubated with the MTT working solution (0.5 mg/mL) for 4 h. Then, add dimethyl sulfoxide reagent (DMSO, Sangon Biotech, Shanghai, China) to the dish to dissolve the violet-colored crystals. Once dissolved, transfer 150 μL of the solution to a 96-well plate for absorbance measurement at 492 nm using a plate reader.

At the same time, spherical cancer cells cultured in 3D were treated with PAM, which was treated by LTP for 0 s, 20 s, and 40 s, and MTT experiments were carried out on cells in different treatment groups, respectively, to compare the differences of plasma sensitivity of A549 cells in 3D culture and 2D culture.

In the 3D culture system based on Cultrex^®^ Basement Membrane Extract (BME, R&D Systems, Minneapolis, MN, USA), we first added 50 μL/well of BME into 96-well plates and incubated them at 37 °C for 1 h to form a reconstituted basement membrane with specific thickness and stiffness. Subsequently, we prepared a cell suspension of A549 cells (1 × 10^4^ cells/200 μL/well) using PAM and seeded it onto the BME-coated plates. After 3 days, we observed the effect of PAM on the size of A549 cell aggregates.

### 4.4. Colony Formation Assay

A549 cells (1000 cells/well) were seeded in culture dishes and observed under a microscope to ensure good adherence and growth after 24 h. The cells were treated with LTP and PAM for 24 h, followed by the replacement with fresh culture medium every 3 days. This cultivation process continued for a period of 7–14 days. After the cells had been fixed and stained, images were acquired. The ratio of colonies developed in the treatment group to those in the control group was used to compute colony formation.

### 4.5. Cell Migration Assay

A549 cells cultured in both 2D and 3D conditions were seeded into 6-well plates and allowed to grow for 24 h until they reached a confluence of over 90%. A straight line was drawn across the cell monolayer using a 10 μL pipette tip, and then the wells were washed with PBS to remove any detached cells and debris. PAM was prepared using serum-free DMEM. The migration of cancer cells was documented at various time points using an inverted microscope. The relative migration rate of A549 cells cultured in 2D and 3D was calculated using ImageJ 1.54f Image J software.

### 4.6. Transwell Invasion Assay

The Matrigel (Corning, Kennebunk, ME, USA) and Transwell system with 8 μm pore polycarbonate filters (Corning, Kennebunk, ME, USA) are utilized for cancer cell invasion assays to establish a chamber for separating highly invasive from less invasive cells. After treating A549 cells with PAM for 4 h, a cell suspension with a concentration of approximately 1 × 10^5^ cells/mL is prepared using DMEM without FBS. Then, 200 μL of this cell suspension is seeded into the upper chamber of the Transwell plate, with the lower chamber containing 500 μL of medium supplemented with 10% FBS. The cells were incubated for 24 h, after which fixation and staining were performed. The stained membranes were photographed under a microscope, and the invading cells were counted.

### 4.7. Extracellular Active Substances Detection in PAM

Extracellular active substances mainly explore the generation of ROS and RNS using an H_2_O_2_ detection kit (Beyotime, Shanghai, China) and a NO detection kit (Beyotime, Shanghai, China), respectively. Add 50 μL of the supernatant from PAM-treated 2D and 3D samples into 96-well plates, followed by the addition of H_2_O_2_ and NO detection reagents for subsequent detection. At the same time, the levels of extracellular ROS and RNS were reassessed after incubation for 4 h. Absorbance at 540 nm was measured, and concentrations were calculated using the standard curve.

### 4.8. Intracellular Active Substances Detection

ROS is the main active substance in cells and was measured in PAM-treated A549 cells after a 4 h incubation using a detection kit (Beyotime, Shanghai, China). Following this, the pre-prepared DCFH-DA probe working solution with a concentration of 10 μM was added, and the cells were further incubated for an additional 30 min. After incubation, PBS was used to clean for 3 times, and 1 mL PBS was added to each dish to observe and record the intracellular ROS production under a fluorescence microscope (DMI8, Leica, Wetzlar, Germany).

### 4.9. NAC Scavenger Assay

N-Acetyl-L-cysteine (NAC, Beyotime, Shanghai, China) was employed as a reagent in the ROS verification experiment, with a working concentration of 10 mM. This experiment aimed to verify whether the alterations in the migration and invasion capabilities of A549 cells induced by PAM were attributable to elevated intracellular ROS levels. Concurrently, cells were pretreated with NAC 1 h before the experiment, followed by the previously specified experimental protocols.

### 4.10. Cell Cycle Detection

A cell cycle detection kit (KGI Biology, Nanjing, China) was used to detect the cell cycle changes of A549 cells after PAM treatment. After the 2D and 3D cultured cells had been incubated with PAM treatment for 24 h, cell suspension was made using trypsin. Following three PBS washes, the cells were transferred to EP tubes. Add 0.3 mL of ice PBS to each tube and mix thoroughly, then add 0.7 mL of anhydrous ethanol. After mixing evenly, put them in a refrigerator at 4 °C and incubate overnight. Centrifuge to obtain the precipitate, then add PBS in each EP tube to wash the cells, and centrifuge to keep the precipitate. In each tube, 0.5 mL of dye solution (RNase/propidium iodide (PI) = 1:9) was added, followed by the analysis of the cell cycle using flow cytometry (BD Biosciences, Franklin Lakes, NJ, USA).

### 4.11. Western Blot

PAM-treated A549 cells were incubated for 24 h, and the cells were lysed with 1% phenylmethylsulfonyl fluoride (PMSF, Beyotime, Shanghai, China) and RIPA (Beyotime, Shanghai, China) lysis buffer. After collecting the lysed cells for mediation and centrifugation, the protein concentration was quantified by using the BCA protein detection kit (Beyotime, Shanghai, China). Then, Western Blot analysis was used, and the SDS-PAGE gel preparation kit (Beyotime, Shanghai, China) was used for gel electrophoresis. Each protein separation channel received 10 μL of sample, and after electrophoresis, the proteins were blotted onto a nitrocellulose membrane (Beyotime, Shanghai, China) to finalize the transfer procedure. After being sealed with 5% skim milk, the primary antibody, which was diluted at a ratio of 1:1000, was added and incubated at 4 °C overnight for more than 12 h. Horseradish peroxidase (HRP, Cell Signaling, Danvers, MA, USA, 1:10,000) as the second antibody was applied and incubated for more than 1 h. Finally, a chemiluminescence kit (Thermo Fisher Scientific, Waltham, MA, USA) and a chemiluminescence gel imaging system (Tanon, Shanghai, China) were used for luminescence imaging. The primary antibodies include rabbit anti-β-Actin antibody (AB Clone, Wuhan, China), rabbit anti-RAS antibody (AB Clone, Wuhan, China), rabbit anti-ERK antibody (Cell Signaling, Danvers, MA, USA), rabbit anti-p-ERK antibody (Cell Signaling, Danvers, MA, USA), rabbit anti-Cyclin D1 antibody (AB Clone, Wuhan, China), rabbit anti-N-Cadherin antibody (Cell Signaling, Danvers, MA, USA), rabbit anti-vimentin antibody (AB Clone, Wuhan, China), rabbit anti-E-Cadherin antibody (AB Clone, Wuhan, China), rabbit anti-MMP-2 antibody (Cell Signaling, Danvers, MA, USA), rabbit anti-MMP-9 antibody (Cell Signaling, Danvers, MA, USA).

### 4.12. Statistical Analysis of Data

The experimental data were presented as mean and standard deviation, and each experiment was conducted three times independently. The experimental data were analyzed by Student’s *t*-test, one-way and two-way ANOVA, and the statistically significant differences were based on * *p* < 0.05, ** *p* < 0.01, *** *p* < 0.001.

## 5. Conclusions

This study has confirmed the anti-cancer effects of PAM on the NSCLC cell line A549 under both 2D and 3D culture conditions. The results demonstrate that PAM treatment inhibits the viability, migration, and invasion capabilities of A549 cells in 2D and 3D cultures, suppresses the EMT process, arrests the cell cycle, and downregulates the expression of the RAS/ERK signaling pathway, which effectively inhibits the formation of tumor spheroids. Furthermore, the effects of PAM on A549 cells are mediated through ROS-induced oxidative reactions, and the malignant behaviors induced by PAM are significantly reversed with NAC treatment. These results indicate that PAM possesses the capacity to suppress the migration of NSCLC cells and the formation of tumor spheroids, thereby offering a theoretical basis for the potential clinical use of PAM in cancer treatment.

## Data Availability

Data are contained within the article.
